# A comprehensive pan-cancer analysis revealing the role of ITPRIPL1 as a prognostic and immunological biomarker

**DOI:** 10.3389/fmolb.2024.1452290

**Published:** 2024-08-15

**Authors:** Wenyuan Duan, Wen Tian, Zhongyi Li, Yunsong Liu, Linping Xu

**Affiliations:** ^1^ Department of Medical Research, The Affiliated Cancer Hospital of Zhengzhou University & Henan Cancer Hospital, Zhengzhou, China; ^2^ Department of Bone and Soft Tissue Cancer, The Affiliated Cancer Hospital of Zhengzhou University & Henan Cancer Hospital, Zhengzhou, China; ^3^ Zhongshan Institute for Drug Discovery, Shanghai Institute of Materia Medica, Chinese Academy of Sciences, Zhongshan, China

**Keywords:** ITPRIP1, The Cancer Genome Atlas, pan-cancer, immune infiltration, therapeutic target

## Abstract

Inositol 1,4,5-Trisphosphate Receptor-Interacting Protein-Like 1 (ITPRIPL1), a single-pass type I membrane protein located in the membrane, functions as an inhibitory ligand of CD3ε. Recent studies have shown that its expression suppresses T cells activation and promote tumor immune evasion. Despite increasing evidence suggesting that ITPRIPL1 plays a significant role in tumor growth, no systematic pan-cancer analysis of ITPRIPL1 has been conducted to date. This study utilized datasets curated from The Cancer Genome Atlas, Genotype Tissue-Expression, and Human Protein Atlas to investigate the relationship between ITPRIPL1 expression and clinical outcomes, immune infiltration, and drug sensitivity across 33 cancer types. We employed multiple methods to assess its prognostic value in pan-cancer, such as univariate Cox regression, survival analysis, and ROC curve analysis and explored the relationship between ITPRIPL1 and tumor mutation burden (TMB), tumor microsatellite instability (MSI), CNV, DNA methylation, immune-related genes, immune cell infiltration, and drug sensitivity to reveal its immunological role. The mRNA expression levels of the ITPRIPL1 gene vary significantly across multiple types of cancer and significantly reduced in breast cancer. Conversely, high ITPRIPL1 expression was associated with a better prognosis in BRCA. Furthermore, the expression of ITPRIPL1 highly correlates with the presence of tumor-infiltrating immune cells and immune checkpoint genes across various types of cancers. Additionally, ITPRIPL1 expression was associated with TMB in 6 cancer types and with MSI in 13 cancer types. High expression of ITPRIPL1 serves as a protective factor in certain cancer types, correlating with longer overall survival in BRCA. Our study further confirms that ITPRIPL1 participates in regulating immune infiltration and affecting the prognosis of patients in pan-cancer. These findings underscore the promising potential of ITPRIPL1 as a therapeutic target for human cancer.

## 1 Introduction

Cancer is a globally public health issue and a notable impediment to prolonging life expectancy due to the escalating incidence of newly identified cases (Sung et al., 2021; Pizzato et al., 2022; Bray et al., 2024). As of now, there is still no definitive method to achieve a complete cure for it. The emergence of immune checkpoint blockade has sparked a paradigm shift in cancer treatment, fundamentally altering the landscape of clinical oncology and signifying a significant departure from traditional methods (Sanmamed and Chen, 2018; Muthukutty et al., 2023). However, while several immune checkpoint blockers, such as PD-1 and PD-L1 antagonists, have been successfully applied, they only offer effective treatment for a small subset of patients with cancer (Wang et al., 2022; Javed et al., 2024). Resistance or limited responsiveness to immunotherapy persists in the treatment of the majority of patients with cancer (Bai et al., 2020; Said and Ibrahim, 2023). It is worth noting that the dysfunction of T cells can significantly impact the effectiveness of immunotherapy, underscoring the profound significance of identifying regulatory molecules associated with this process (Xia et al., 2019; Zhang et al., 2020; Zheng et al., 2021; Oliveira and Wu, 2023).

Inositol 1,4,5-triphosphate receptor-interacting protein-like 1 (ITPRIPL1), a single-pass transmembrane protein, functions as a natural ligand of CD3ε, leading to the reduction of T cell activity and fostering tumor growth (Deng et al., 2024). Recent studies have found that ITPRIPL1 is commonly observed in tumors with low expression of PD-L1 and the expression of this gene can inhibit T cells in the tumor microenvironment (TME), while antibodies targeting this gene can suppress tumor growth and promote T cell infiltration (Deng et al., 2023; Deng et al., 2024). These findings indicate that ITPRIPL1 holds promise as a new therapeutic target. However, research on ITPRIPL1, particularly exploring its potential roles in various human cancer types using multi-omics data, is currently inadequate. A comprehensive analysis of its functional roles across multiple malignant tumors using pan-cancer analysis is imperative.

In this study, we employed utilized a range of bioinformatics methodologies to perform a comprehensive pan-cancer analysis of ITPRIPL1 from various perspectives. Multiple databases, including The Cancer Genome Atlas (TCGA), Human Protein Atlas (HPA) and Genotype-Tissue Expression (GTEx) were used to explore the mRNA expression levels of ITPRIPL1 in tumor and normal tissues (Weinstein et al., 2013; Carithers et al., 2015; GTEx Consortium, 2020; Sjöstedt et al., 2020). We employed the Gene Set Cancer Analysis (GSCA) database to analyze the potential associations between ITPRIPL1 expression and Genomic alterations and DNA methylation across 33 types of cancer (Liu et al., 2023). We also did univariate Cox regression analysis and survival analysis, receiver operating characteristic (ROC) curve analysis, Immune infiltration analysis, and Drug sensitivity analysis. Furthermore, we computed the correlation of ITPRIPL1 expression with tumor mutation burden (TMB), microsatellite instability (MSI), and immune-related genes. Based on all these results, we unveiled a correlation between ITPRIPL1 expression and immune response, suggesting its potential as a promising prognostic biomarker across various cancers. Our research endeavors to offer further elucidation on the significance of ITPRIPL1 across different cancer types.

## 2 Materials and methods

### 2.1 ITPRIPL1 expression analysis

The bulk RNA-seq data of all 33 cancer types in TCGA and normal samples were obtained from the UCSC Xena (http://xena.ucsc.edu/), HPA (https://www.proteinatlas.org/) and GTEx (http://commonfund.nih.gov/GTEx/) projects (Weinstein et al., 2013; Carithers et al., 2015; Goldman et al., 2020; GTEx Consortium, 2020; Sjöstedt et al., 2020). The mRNA expression levels of ITPRIPL1 in pan-cancer and different tissues were analyzed. And we calculated the differential expression of ITPRIPL1 based on clinical stage.

### 2.2 Methylation and CNV analysis

GSCA(https://guolab.wchscu.cn/GSCA/#/) is an integrated bioinformatics analysis platform (Liu et al., 2023). We selected the “Mutation” module to analysis CNVs and methylation of ITPRIPL1 and correlation with mRNA expression levels for all cancers. The “CNV & Expression” module calculates the Spearman correlation between RSEM-normalized mRNA expression and CNV data from the TCGA database, with *p*-values adjusted by FDR. The “Differential Methylation” module provides analysis between tumor and normal sample groups using Illumina HumanMethylation 450k level 3 data from the TCGA database, selecting 14 cancer types with over 10 paired samples, filtering the most negatively correlated methylation sites with gene expression, and estimating *p*-values by t-test adjusted by FDR. The “Methylation & Expression” module analyzes the correlation between methylation levels and mRNA expression using RSEM-normalized mRNA and Illumina Methylation 450k level 3 data from the TCGA database, merging data by TCGA barcode, filtering for the most negatively correlated methylation sites, and performing Spearman correlation with *p*-values adjusted by FDR.

### 2.3 Univariate cox regression and survival analysis

Univariate Cox regression was employed to analysis the statistical significance of overall survival (OS), disease-specific survival (DSS), disease-free interval (DFI), and progression-free interval (PFI) between high and low ITPRIPL1 expression groups across 33 cancer types, with statistical significance set at *P* < 0.05 (Liu et al., 2018). Furthermore, Kaplan-Meier survival analysis was used to investigate the prognostic value of ITPRIPL1 with 33 cancer types in TCGA. ITPRIPL1 expression level above the median are considered high expression, while those below are considered low expression. The “survminer” (version 0.4.9) and “survival” (version 3.5.7) packages were used for bioinformatics analysis based on the R language.

### 2.4 ROC curve analysis

We conducted ROC curve analysis and evaluated the diagnostic value of the ITPRIPL1 gene, using the “pROC” package (version 1.18.5), where an AUC value greater than 0.7 was considered helpful for disease screening (Robin et al., 2011).

### 2.5 Immune infiltration analysis

To comprehensively delineate the tumor microenvironment and immune landscape of various cancers, the CIBERSORT, MCPcounter, and ssGSEA methods were employed to examine the relationship between ITPRIPL1 expression and the infiltration abundance of various immune cells in various cancers (Becht et al., 2016; Chen et al., 2018; Yi et al., 2020). For each patient, the estimation of stromal and immune cells in malignant tumors using expression data (ESTIMATE) method was used to calculate the stromal score, immune score and estimate score to infer tumor purity using the R package “estimate” (version 1.0.13) (Yoshihara et al., 2013).

### 2.6 Correlation of ITPRIPL1 expression with tumor mutation burden, tumor microsatellite instability and immune-related genes

MSI refers to the results of changes in the length of microsatellite sequences caused by insertions or deletions mutations during DNA replication (Yang et al., 2019; Li et al., 2020). The MSI analysis utilized the “MSIsensor10k” dataset within the “BiocOncoTK” package (version 1.22.2), and Spearman’s method was employed to compute the correlation with the ITPRIPL1 gene. The “TCGAmutations” package (version 0.3.0) was used to download mutation data containing all samples from TCGA and the TMB of samples was calculated using the “tmb” function from the “maftools” package (version 2.18.0). Correlation analysis between the expression of ITPRIPL1 and TMB was performed using Spearman’s method. We conducted an expression correlation analysis between ITPRIPL1 and immune-related genes using the Spearman’s method, including genes encoding major histocompatibility complex (MHC), Mismatch Repair (MMR), immune checkpoints, chemokines, and chemokine receptor proteins.

### 2.7 Drug sensitivity analysis

We selected the “Drug” module to analysis the correlation between ITPRIPL1 gene expression and drug sensitivity across all cancers (Liu et al., 2023). Pearson correlation analysis was performed to get the correlation between ITPRIPL1 expression and drug IC50, using data collected from the Genomics of Drug Sensitivity in Cancer (GDSC) and the Genomics of Therapeutics Response Portal (CTRP) (Basu et al., 2013; Yang et al., 2013).

### 2.8 Statistical analysis

All gene expression data were normalized using log2 transformation. The comparison between normal tissue and cancer tissue was conducted using two sets of Wilcoxon tests, with statistical significance indicated by *P* < 0.05. Spearman’s test was employed for correlation analysis between variables, with *P* < 0.05 considered significant. All statistical analyses were performed using R software (version 4.3.1).

## 3 Results

### 3.1 ITPRIPL1 expression in various human normal and tumor tissues

**FIGURE 1:**
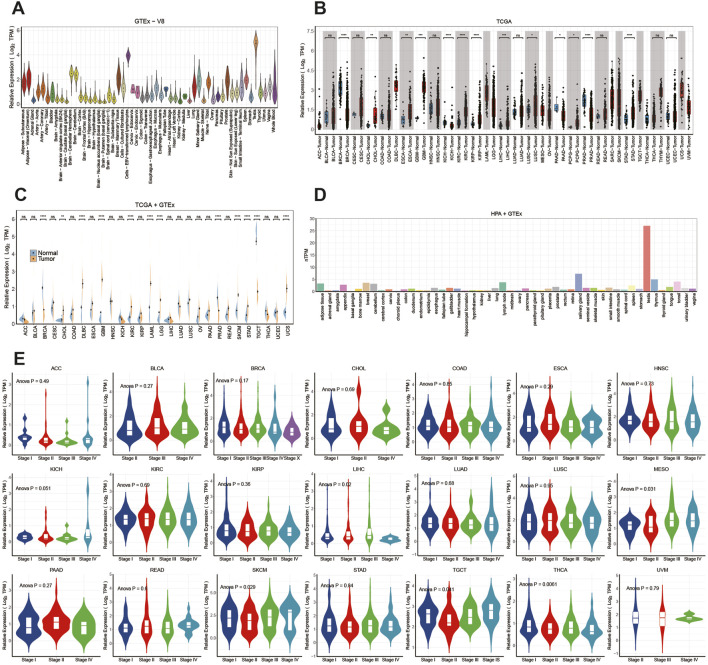
The differential expression of ITPRIPL1 in pan-cancer and healthy tissues: **(A)** Distribution of ITPRIPL1 expression from the GTEx database. **(B)** Analysis of ITPRIPL1 expression in tumor and normal tissues curated from the TCGA database. **(C)** Analysis of ITPRIPL1 expression in tumor and normal tissues in the TCGA and GTEx databases. **(D)** Analysis of ITPRIPL1 expression in the HPA and GTEx databases. **(E)** ITPRIPL1 expression levels across different stages in pan-cancer. (ns: *p* > 0.05, ^*^: P ≤ 0.05, ^**^: P ≤ 0.01, ^***^: P ≤ 0.001, ^****^: P ≤ 0.0001.). BRCA (Breast Cancer), UCEC (Uterine Corpus Endometrial Carcinoma), KIRC (Kidney Renal Clear Cell Carcinoma), HNSC (Head and Neck Squamous Cell Carcinoma), LUAD (Lung Adenocarcinoma), LGG (Low Grade Glioma), THCA (Thyroid Cancer), LUSC (Lung Squamous Cell Carcinoma), PRAD (Prostate Adenocarcinoma), SKCM (Cutaneous Melanoma), COAD (Colorectal Adenocarcinoma), OV (Ovarian Cancer), STAD (Stomach Adenocarcinoma), BLCA (Bladder Urothelial Carcinoma), LIHC (Liver Hepatocellular Carcinoma), CESC (Cervical Squamous Cell Carcinoma and Endocervical Adenocarcinoma), KIRP (Kidney Renal Papillary Cell Carcinoma), SARC (Sarcoma), ESCA (Esophageal Carcinoma), PCPG (Pheochromocytoma and Paraganglioma), PAAD (Pancreatic Adenocarcinoma), GBM (Glioblastoma Multiforme), READ (Rectal Adenocarcinoma), LAML (Acute Myeloid Leukemia), TGCT (Testicular Germ Cell Tumor), THYM (Thymoma), MESO (Malignant Mesothelioma), UVM (Uveal Melanoma), ACC (Adrenocortical Carcinoma), KICH (Kidney Chromophobe), USC (Uterine Carcinosarcoma), DLBC (Diffuse Large B-cell Lymphoma), CHOL (Cholangiocarcinoma).

RNA-seq datasets downloaded from HPA, TCGA and GTEx databases were employed to explore ITPRIPL1 expression in various human normal and tumor tissues. As depicted in Figures 1A, D, the tissue exhibiting the highest ITPRIPL1 expression was the testis. The consensus transcript expression levels of the ITPRIPL1 gene across 50 tissues are summarized based on transcriptomics data from HPA and GTEx (Figure 1D). Through analysis of TCGA data, we showed the expression levels of the ITPRIPL1 gene from a pan-cancer perspective and found statistically significant differences in expression levels between tumor tissues and correspond normal tissues across 13 common malignancies (Figure 1B). The mRNA expression levels of the ITPRIPL1 gene was significantly increased in cholangiocarcinoma (CHOL), esophageal carcinoma (ESCA), glioblastoma multiforme (GBM), kidney renal clear cell carcinoma (KIRC), kidney renal papillary cell carcinoma (KIRP), liver hepatocellular carcinoma (LIHC), lung squamous cell carcinoma (LUSC), pheochromocytoma and paraganglioma (PCPG), stomach adenocarcinoma (STAD) and reduced in breast cancer (BRCA), kidney chromophobe (KICH), prostate adenocarcinoma (PRAD), pancreatic adenocarcinoma (PAAD).

Expanding our analysis, we enriched the pool of normal samples by integrating data from the GTEx database. This meticulous approach unveiled statistically significant differences in expression across 12 cancer types (Figure 1C). The mRNA expression levels of the ITPRIPL1 gene were found to be elevated in CHOL, diffuse large B-cell lymphoma (DLBC), GBM, KIRC, acute myeloid leukemia (LAML), lower grade glioma (LGG), skin cutaneous melanoma (SKCM), STAD, uterine carcinosarcoma (UCS), while reduced in BRCA, PRAD and Testicular Germ Cell Tumors (TGCT).

Furthermore, using the “TCGAbiolinks” package (version 2.30.4), we retrieved clinical stages and other clinical data of all patients from The TCGA database. We observed the mRNA expression levels of ITPRIPL1 varied significantly across clinicopathological stages in mesothelioma (MESO), LIHC, SKCM, thyroid carcinoma (THCA), and TGCT (Figure 1E) (*P* < 0.05). However, in other tumors, there was no significant difference observed (*P* > 0.05) (Figure 1E).In MESO, we observed that the mRNA expression levels of ITPRIPL1 gradually increased from stage I to stage IV, suggesting its potential role in tumor progression. Data from SKCM showed that ITPRIPL1 expression was lower in early stages (I and II) but higher in stages IV. Finally, in TGCT, ITPRIPL1 expression was lower in stages II but higher in stages IV. However, in other tumor types, we did not observe significant differences in ITPRIPL1 mRNA expression levels across different clinicopathological stages (*P* > 0.05). These results suggest that ITPRIPL1 expression levels, which vary significantly across different clinicopathological stages in certain cancer types, may serve as a new biomarker for diagnosis and staging specific cancers.

### 3.2 Methylation and CNV profile of ITPRIPL1 in pan-cancer

**FIGURE 2:**
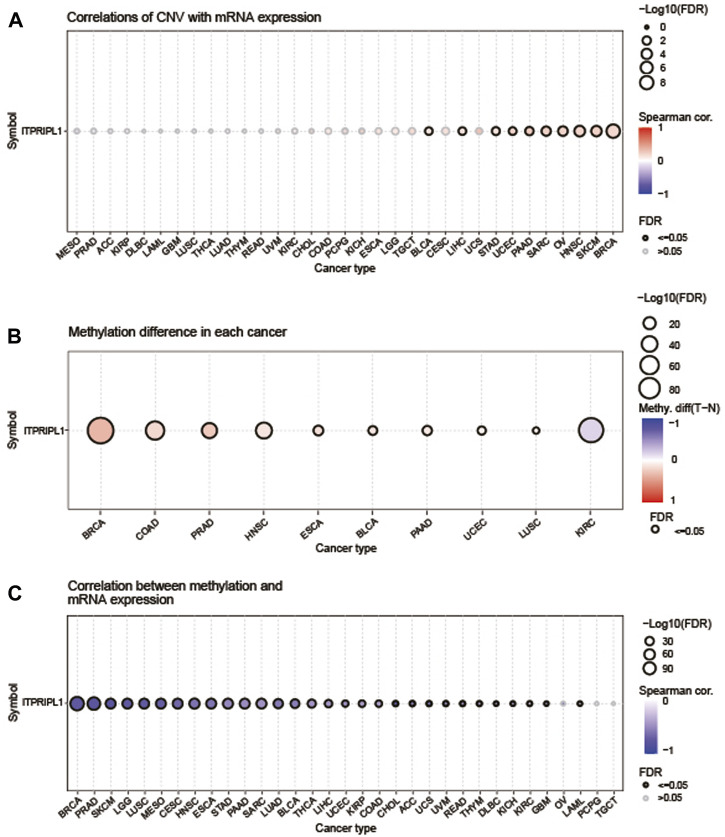
Methylation and CNV Analysis. **(A)** The correlation between CNV and ITPRIPL1 mRNA expression was analyzed using the GSCA database. The highest correlation was observed in PAAD at 0.24. The bubble plot depicts the correlation between ITPRIPL1 mRNA expression and CNV levels: blue bubbles denote negative correlations, while red ones denote positive correlations, with intensity reflecting strength. Bubble size indicates FDR significance, and a black outline signifies FDR ≤0.05. **(B)** This figure summarizes the differences in methylation between tumor and normal samples for ITPRIPL1 across various cancers. **(C)** The correlation between methylation and ITPRIPL1 mRNA expression was analyzed using the GSCA database.

We utilized the GSCA database to examine the correlation between ITPRIPL1 expression levels and CNV and methylation in pan-cancer. Of all the statistically significant results, the highest correlation was found in PAAD at 0.24, followed by sarcoma (SARC) and SKCM, both at 0.22 (Figure 2A). Conversely, the correlations were not significant in patients with MESO, PRAD, adrenocortical carcinoma (ACC), KIRP, DLBC, LAML, GBM, LUSC, THCA, lung adenocarcinoma (LUAD), thymoma (THYM), rectum adenocarcinoma (READ), uveal melanoma (UVM), KIRC, CHOL, colon adenocarcinoma (COAD), PCPG, KICH, ESCA, LGG, TGCT, cervical squamous cell carcinoma and endocervical adenocarcinoma (CESC), and UCS (Figure 2A). We also employed the GSCA database to explore the methylation and its correlation with ITPRIPL1 expression. The “Differential Methylation” module was selected for conducting differential methylation analysis between tumor and normal sample groups. Among all the analyzed cancer types, only KIRC showed a downward trend, while others exhibited an upward trend (Figure 2B). And we found that DNA methylation levels were significantly correlated with mRNA expression in most tumor types (Figure 2C). The DNA methylation levels exhibited significant correlations with mRNA expression levels across various cancer types and the top three tumors with the most significant correlations were CHOL (Spearman’s correlation = −0.77), PRAD (Spearman’s correlation = −0.77), and SKCM (Spearman’s correlation = −0.78), showing a robust negative correlation close to −0.8 with significant statistical significance (*P* < 0.05).

### 3.3 Prognostic value of ITPRIPL1 in pan-cancer

**FIGURE 3:**
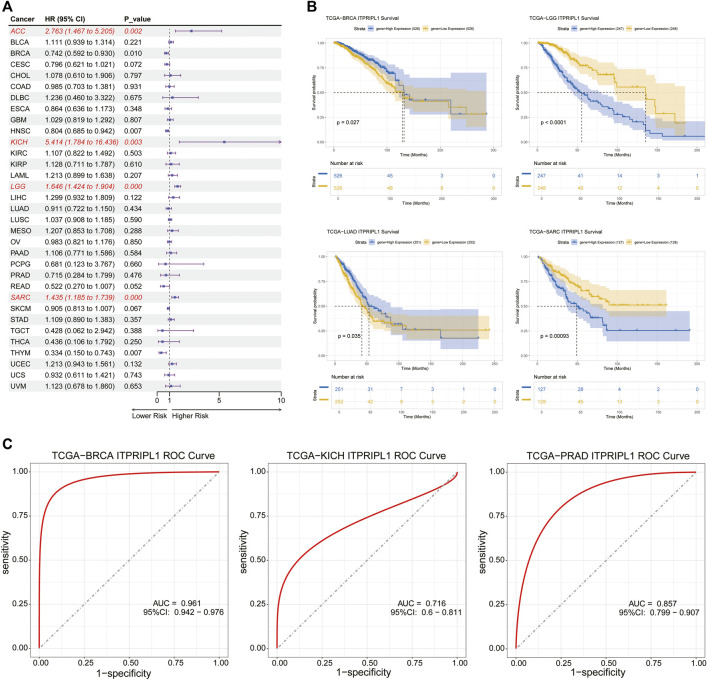
**(A)** The relationship between ITPRIPL1 expression and overall survival (OS) in 33 cancer types was analyzed using univariate Cox regression analysis. **(B)** Kaplan–Meier survival curve analysis of ITPRIPL1 in BRCA, LGG, LUAD, and SARC. **(C)** Employed the ROC curve analysis to evaluate the performance of ITPRIPL1 in BRCA, KICH, and PRAD.

Univariate cox regression analysis was utilized to explore the association between OS, DSS, DFI and PFI and the mRNA expression levels of ITPRIPL1 in pan-cancer. High expression of ITPRIPL1 was strongly associated with shorter OS in ACC (OS: HR = 2.763, *P* = 0.002), KICH (OS: HR = 5.414, *P* = 0.003), LGG (OS: HR = 1.646, *P* = 0.000) and SARC (OS: HR = 1.435, *P* = 0.000), but was a protective factor for BRCA (OS: HR = 0.742, *P* = 0.010), head and neck squamous cell carcinoma (HNSC) (OS: HR = 0.804, *P* = 0.007) and THYM (OS: HR = 0.334, *P* = 0.007) (Figure 3A). ITPRIPL1 was also a high-risk gene for DFI, DSS and PFI in SARC, a risk factor for DSS and PFI in ACC, KICH and LGG and a risk factor for PFI in UCEC, but it was a protective factor for DFI in ESCA and for DSS and PFI in BRCA, HNSC (Supplementary Figures S1, 2, 3). In conclusion, these results indicated that high expression of ITPRIPL1 was highly associated with poor prognosis in ACC, KICH, LGG, and SARC. Furthermore, Kaplan-Meier survival analysis showed that high expression of ITPRIPL1 had longer survival times with in BRCA and LUAD (Figure 3B). In contrast, high ITPRIPL1 expression levels predicted poor prognosis in LGG and SARC (Figure 3B). Interestingly, these results indicated that ITPRIPL1 expression levels are strongly correlated with prognosis in some cancers like LGG. We performed ROC analysis and calculated the AUC values of the ITPRIPL1 gene, plotting the ROC curves for the three cancers with high accuracy (AUC >0.7), specifically: BRCA (AUC = 0.961,CI 0.942–0.976), KICH (AUC = 0.716,CI 0.6–0.811), and PRAD (AUC = 0.857,CI 0.799–0.907) (Figure 3C).

### 3.4 Correlation between ITPRIPL1 expression and immune infiltrating level and immune-related genes in pan-cancer

**FIGURE 4:**
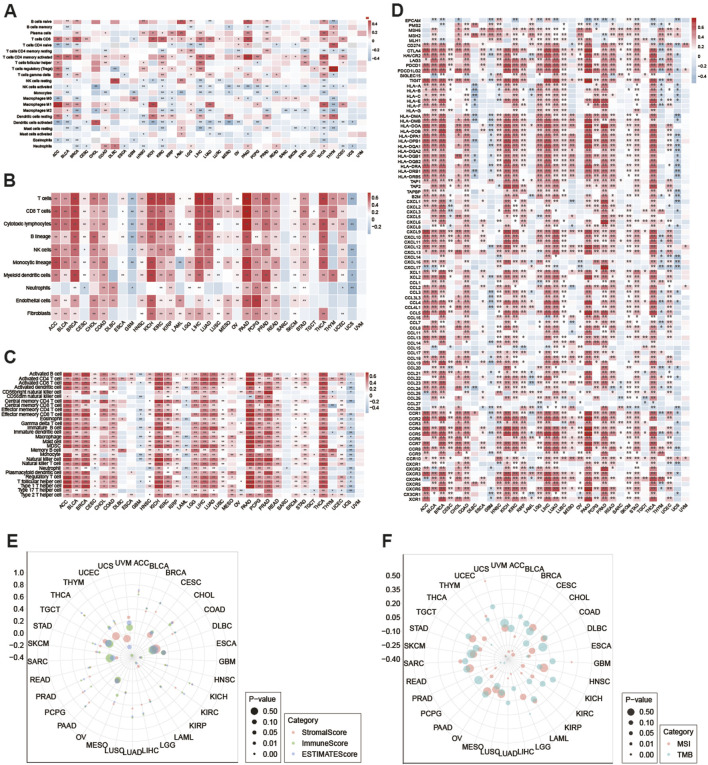
**(A)** Using the CIBERSORT method to calculate the correlation between ITPRIPL1 mRNA expression and 22 types of immune cells across various cancers. (^*^: *P* < 0.05, ^**^: *P* < 0.01). Using **(B)** MCPcounter and **(C)** ssGSEA methods to examine the relationship between ITPRIPL1 expression and the infiltration abundance of various immune cells in various cancers. Drug Sensitivity Analysis. **(D)** Performed a Spearman correlation analysis between ITPRIPL1 and immune-related genes, including those encoding major histocompatibility complex, mismatch repair, immune checkpoints, chemokines, and chemokine receptors. (^*^: *P* < 0.05, ^**^: *P* < 0.01). **(E)** Using the ESTIMATE algorithm to assess the correlation of ITPRIPL1 expression with stroma score, immune score and estimate score. **(F)** Radar maps showed the correlation between ITPRIPL1 expression with TMB and MSI in pan-cancer.

Using the CIBERSORT, MCPcounter, and ssGSEA algorithms, we explored the relationship between ITPRIPL1 expression and the infiltration levels of different immune cells in the 33 cancer types. In the CIBERSORT analysis results, we observed that the expression level of ITPRIPL1 is positively correlated with activated memory CD4^+^ T cells in PAAD (Spearman r = 0.486, *P* = 6.341E-12), regulatory T cells (Tregs) in THYM (Spearman r = 0.530, *P* = 5.664E-10), and Macrophage (M1) in ACC (Spearman r = 0.499, *P* = 2.896E-06) and negatively associated with activated NK cells in CHOL (Spearman r = −0.451, *P* = 0.006) and activated dendritic cells in MESO (Spearman r = −0.455, *P* = 1.080E-05) (Figure 4A). By employing the MCPcounter algorithm, we observed that the expression level of ITPRIPL1 shows a strong positive correlation with T cells in LIHC, as well as with T cells, CD8^+^ T cells, and cytotoxic lymphocytes in PAAD, with Spearman r values greater than 0.7 and *P*-values less than 0.001 from the MCPcounter analysis results and negative correlations with a Spearman r value less than −0.4 were not observed (Figure 4B). To evaluate the infiltration levels of different immune cells, the ssGSEA algorithm was employed. The expression level of ITPRIPL1 is strongly positively correlated with various immune cells, including activated CD8^+^ T cells, CD56bright natural killer cells, effector memory CD8^+^ T cells, natural killer cells, T follicular helper cells, type 1 T helper cells, and myeloid-derived suppressor cells (MDSCs) across multiple types of cancer, particularly in PAAD with *P* < 0.001 and Spearman r value greater than 0.7 for all (Figure 4C). The results indicate that ITPRIPL1 might be crucial in controlling immune cell infiltration in some cancer types. Given the significant influence of immune checkpoint-related genes on immune cell infiltration and immunotherapy, we explored the potential role of ITPRIPL1 in immunotherapy by examining its expression levels in relation to immune checkpoint-related genes and other key associated genes in human cancers. Our results showed that ITPRIPL1 expression was associated with most genes in ACC, BRCA, KICH, LIHC, LUAD, PAAD, PRAD, THCA, and DLBC with *P* < 0.001(Figure 4D). We found strong positive correlations between ITPRIPL1 expression and three types of ESTIMATE in LUAD, PAAD, LIHC, BRCA, CHOL, KIRC, THCA, KICH, ACC, and PCPG, whereas negative correlations were observed in LAML, SARC, UCS and GBM (Figure 4E).

### 3.5 Correlation analysis of ITPRIPL1 with TMB and MSI in TCGA pan-cancer

In addition, we also calculated the TMB and MSI expression. ITPRIPL1 expression showed a positive correlation with MSI in patients with GBM, UCEC, and UCS, with *P* < 0.05. And ITPRIPL1 expression exhibited a negative correlation with MSI in patients with ACC, BLCA, BRCA, ESCA, KIRP, LIHC, LUAD, PCPG, PRAD, and THYM, with *P* < 0.05 (Figure 4F). Additionally, ITPRIPL1 expression was positively correlated with TMB in LGG and negatively correlated with TMB in ESCA, PAAD, PRAD, THCA and THYM with *P* < 0.05 (Figure 4F).

### 3.6 Correlations between ITPRIPL1 and drugs

**FIGURE 5:**
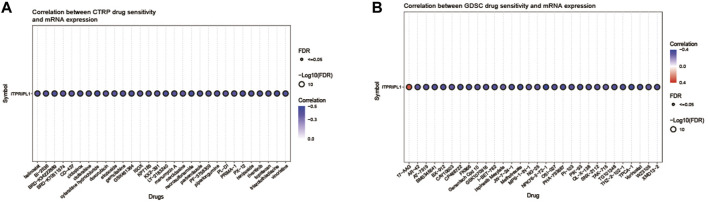
**(A)** This figure summarizes the correlation between gene expression and the sensitivity to the CTRP drugs (top 30) in pan-cancer. **(B)** This figure summarizes the correlation between gene expression and the sensitivity to the GDSC drugs (top 30) in pan-cancer.

Using the GSCA online platform, we explored and presented the correlation between ITPRIPL1 gene expression and sensitivities of the top 30 drugs, utilizing data curated from the GDSC and CTRP databases. The CTRP and GDSC data analysis revealed that apart from 17-AAG, drug sensitivity and ITPRIPL1 gene expression were negatively correlated for the remaining 59 drugs. (Figures 5A, B).

## 4 Discussion

There are many existing cancer treatments, among which tumor immunotherapy is a promising anti-cancer strategy (Peterson et al., 2022; Singh et al., 2024). However, due to the significant variability in treatment efficacy among different patients, identifying potential therapeutic targets to enhance treatment effectiveness has been a focal point of interest (Kim et al., 2019; Kudo, 2020). One recent study has identified the ITPRIPL1 gene as a potential new immune checkpoint that binds with CD3ε, blocking T cell activation and facilitating immune evasion by tumors (Deng et al., 2024). This study conducted a comprehensive pan-cancer analysis of ITPRIPL1 expression by integrating data from multiple databases, including TCGA, GTEx, and HPA, through various bioinformatics analysis methods.

In this study, we analyzed public data from HPA, TCGA, and GTEx and showed the expression of ITPRIPL1 in various human normal and tumor tissues. The mRNA expression levels of ITPRIPL1 were significantly increased in CHOL, ESCA, GBM, KIRC, KIRP, LIHC, LUSC, PCPG, and STAD, while reduced in BRCA, KICH, PRAD, and PAAD. Expanding our analysis by integrating additional normal samples from the GTEx database, we found that ITPRIPL1 mRNA expression levels were elevated in CHOL, DLBC, GBM, KIRC, LAML, LGG, SKCM, STAD, and UCS, but reduced in BRCA, PRAD, and TGCT. We observed that the ITPRIPL1 expression varied significantly across MESO, LIHC, SKCM, THCA, and TGCT. We examined the correlation between ITPRIPL1 expression levels and CNV and methylation patterns in pan-cancer using the GSCA database, finding the highest correlations in PAAD and BRCA, with DNA methylation levels showing significant correlations with mRNA expression across various cancer types, particularly in CHOL, PRAD, SKCM, and BRCA. ITPRIPL1 may play various roles in cancer prognosis. Univariate Cox regression analysis revealed that high ITPRIPL1 mRNA expression is strongly associated with shorter OS in ACC, KICH, LGG, and SARC, but serves as a protective factor in BRCA, HNSC, and THYM. Additionally, ITPRIPL1 is a high-risk gene for DFI, DSS, and PFI in SARC, and a risk factor for DSS and PFI in ACC, KICH, and LGG, while acting as a protective factor for DFI in ESCA and for DSS and PFI in BRCA and HNSC. Kaplan-Meier survival analysis indicated that high ITPRIPL1 expression is associated with longer survival in BRCA and LUAD, but predicts poor prognosis in LGG and SARC. ROC analysis showed high accuracy (AUC >0.7) for ITPRIPL1 in BRCA, KICH, and PRAD. The results from different analyses show some consistency, particularly in the cancer types where ITPRIPL1 expression is either elevated or reduced and its impact on prognosis. However, there are discrepancies, such as its dual role as a risk and protective factor in different cancer types, indicating a complex, context-dependent function of ITPRIPL1 in tumor biology.

TME is a complex ecosystem including diverse immune cells, cancer cells, and other components and is critical for cancer proliferation, invasion, and metastasis (Giraldo et al., 2019; Anderson and Simon, 2020; Neophytou et al., 2021). Given the essential role of Immune cells is responsible for eliminating tumor cells, with their infiltration density and activity shown to predict responses to immune checkpoint blockers and serve as independent prognostic markers for cancer patients (Goubet et al., 2023; Huang et al., 2024; Ock et al., 2016). We employed the CIBERSORT, MCPcounter, and ssGSEA algorithms to explore the relationship between ITPRIPL1 expression and the infiltration levels of different immune cells in the 33 cancer types. We observed the ITPRIPL1 expression is strongly associated with activated memory CD4^+^ T cells, CD8^+^ T cells, regulatory T cells (Tregs), M1 macrophages, NK cells and activated dendritic cells in some cancer types. These findings suggest that ITPRIPL1 may play a significant role in regulating immune cell infiltration in various cancer types. Considering the impact of immune checkpoint-related genes on immune cell infiltration and immunotherapy, we also conducted relevant analysis. Our results showed that ITPRIPL1 expression is associated with most genes in ACC, BRCA, KICH, LIHC, LUAD, PAAD, PRAD, THCA, and DLBC, indicating its potential role in immunotherapy. Using the ESTIMATE algorithm to assess the stromal and immune score across 33 cancer types, we found that ITPRIPL1 expression is strongly positively correlated with these scores in LUAD, PAAD, LIHC, BRCA, CHOL, KIRC, THCA, KICH, ACC, and PCPG, while negative correlations were observed in LAML and GBM. Obviously, our results showed that ITPRIPL1 is associated with responses to immunotherapy.

Recent research has reported that ITPRIPL1 is a promising therapeutic target (Deng et al., 2023; Deng et al., 2024). The extracellular domain of ITPRIPL1 binds to CD3ε on T cells, significantly reducing calcium influx and ZAP70 phosphorylation, thereby hindering initial T cell activation (Deng et al., 2024). MSI and TMB are major biomarkers used to identify potential benefit from immune checkpoint inhibitors for patients, which are powerful predictors of tumor behavior and response to immunotherapy (Li et al., 2020; Palmeri et al., 2022). Here, we evaluated the correlation between ITPRIPL1 expression, TMB, and MSI. We observed that ITPRIPL1 expression was associated with TMB in 6 cancer types and with MSI in 13 cancer types.

In summary, our findings further confirmed that ITPRIPL1 holds promise as a prognostic biomarker across various cancers and as a predictive indicator for immunotherapy. These results contribute to our comprehension of the potential impact of ITPRIPL1 on tumor immunity and its relevance to strategies for immunotherapy. However, the study has limitations, such as the reliance on public databases which may contain biases, and the need for experimental validation to confirm the bioinformatics predictions. Future research should focus on these aspects to fully elucidate the role of ITPRIPL1 in cancer biology and therapy.

## Data Availability

The original contributions presented in the study are included in the article/Supplementary Material, further inquiries can be directed to the corresponding authors.
