# Supplementary material for: A comprehensive pan-cancer analysis revealing the role of ITPRIPL1 as a prognostic and immunological biomarker
Source: Front Mol Biosci. 2024 Aug 15;11:1452290. doi: 10.3389/fmolb.2024.1452290 (PMC11357910; doi:10.3389/fmolb.2024.1452290)
Supplement: Supplementary file 1 [file DataSheet1.DOCX]

Supplementary Material

# Supplementary Figures


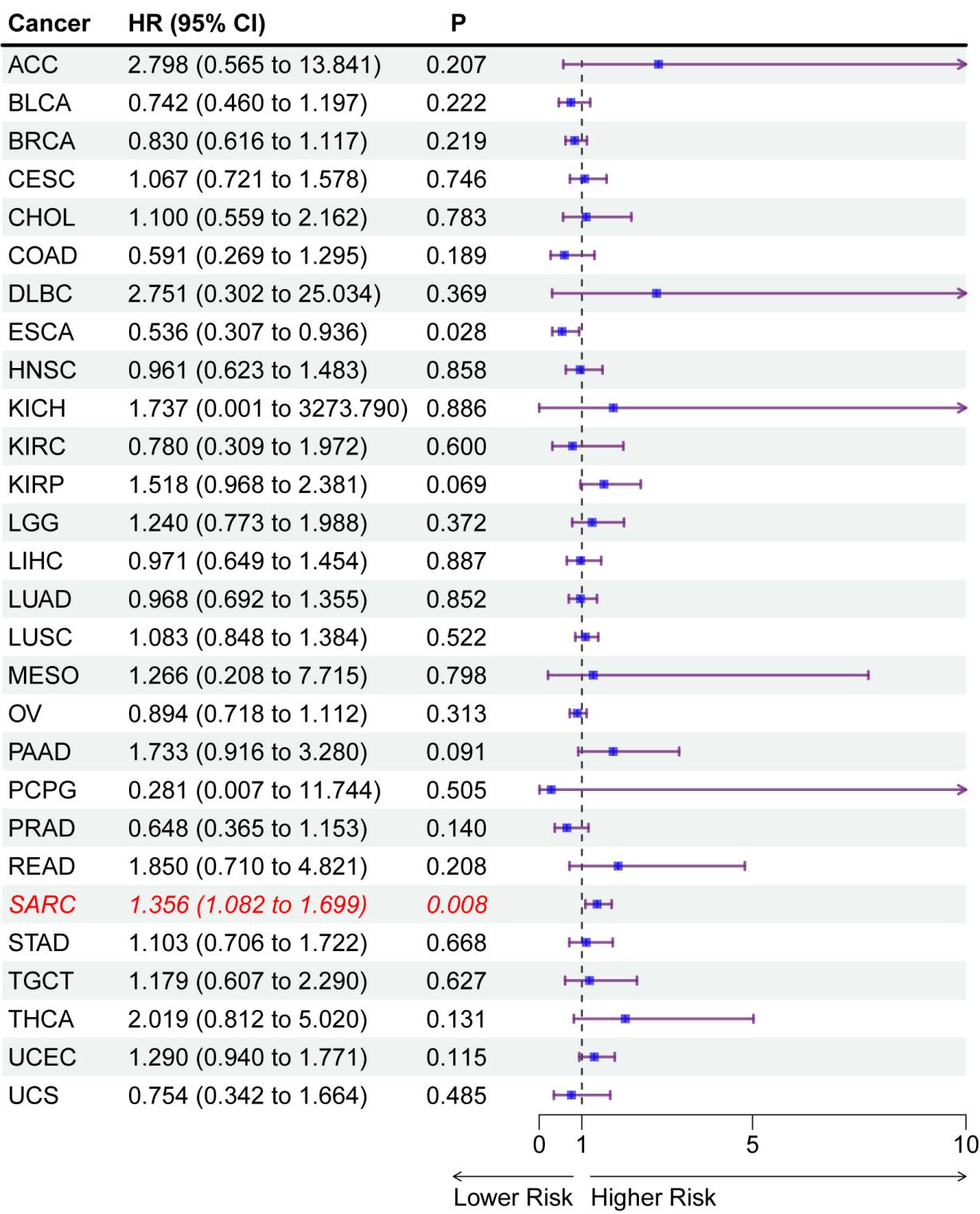


**Supplementary Figure 1.** Performing univariate Cox regression to investigate the association between DFI and the mRNA expression levels of ITPRIPL1 in pan-cancer.


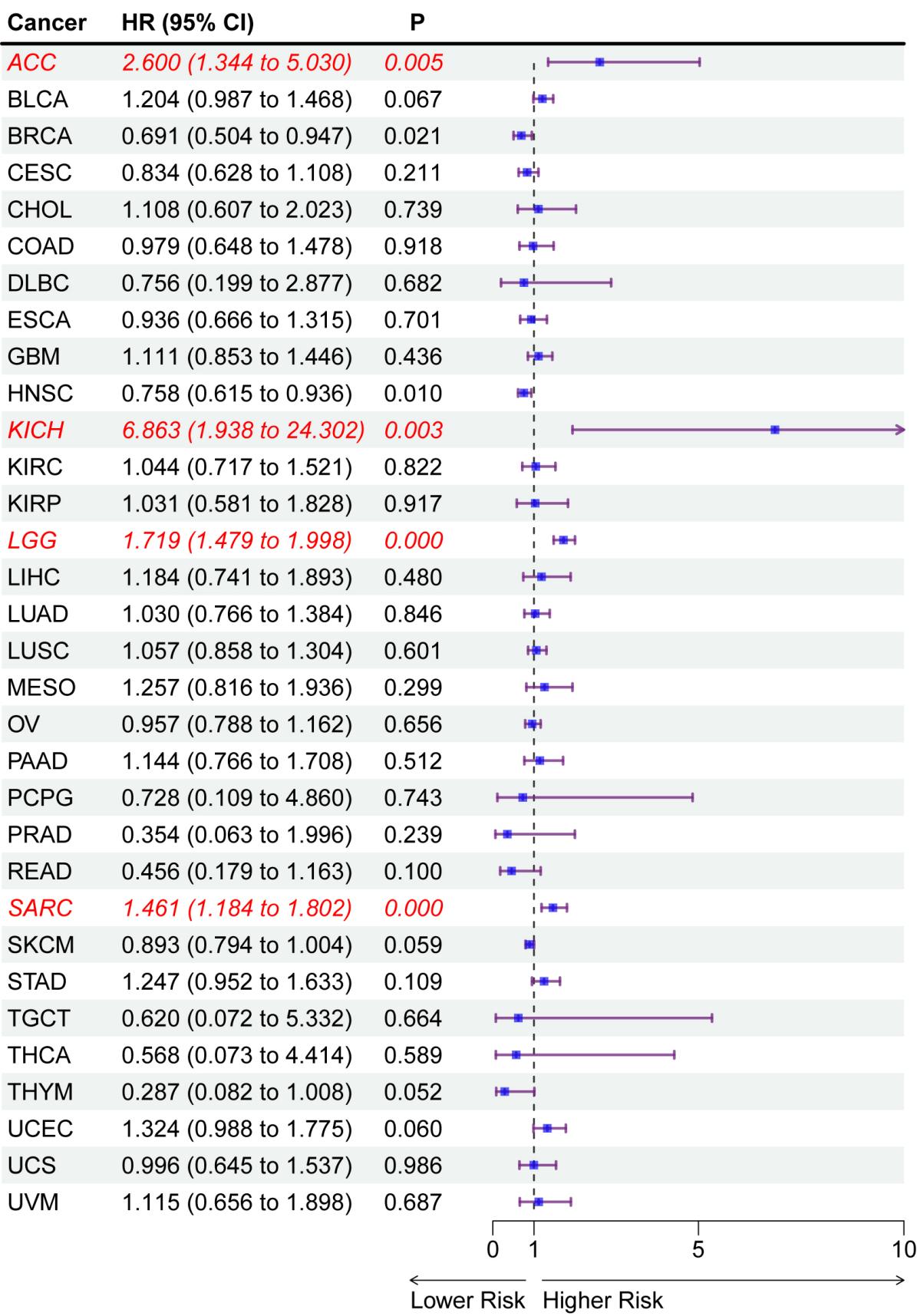


**Supplementary Figure 2.** Performing univariate Cox regression to investigate the association between DSS and the mRNA expression levels of ITPRIPL1 in pan-cancer.


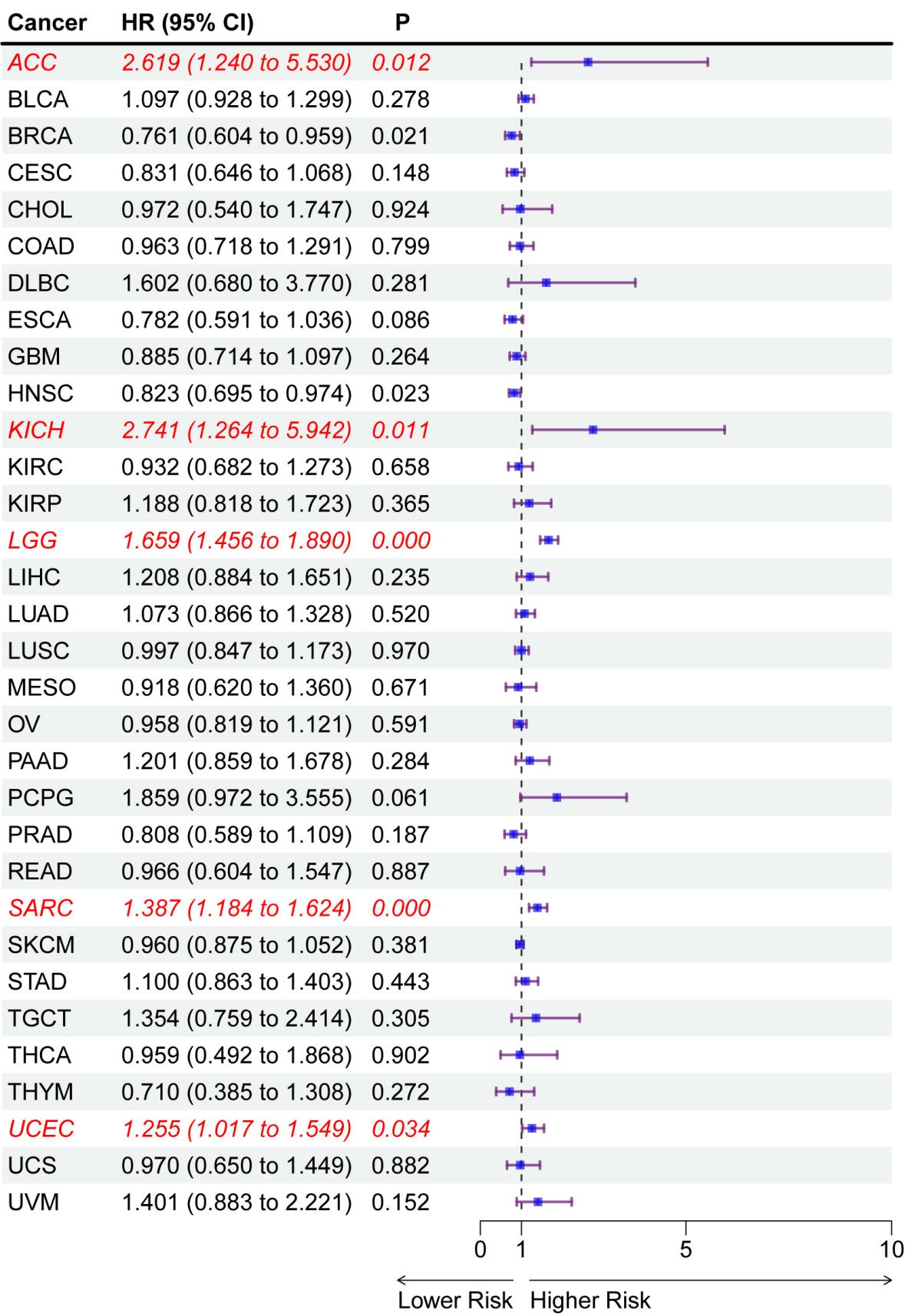


**Supplementary Figure 3.** Performing univariate Cox regression to investigate the association between PFI and the mRNA expression levels of ITPRIPL1 in pan-cancer.
